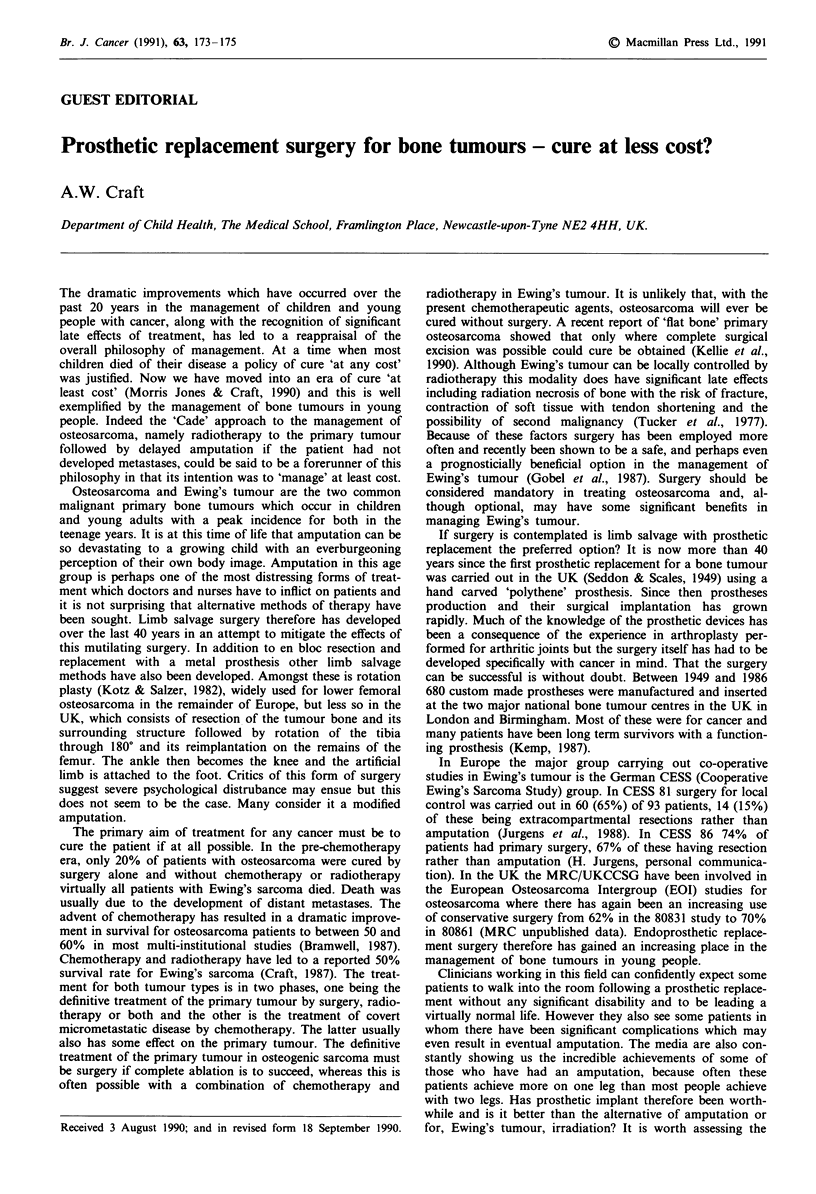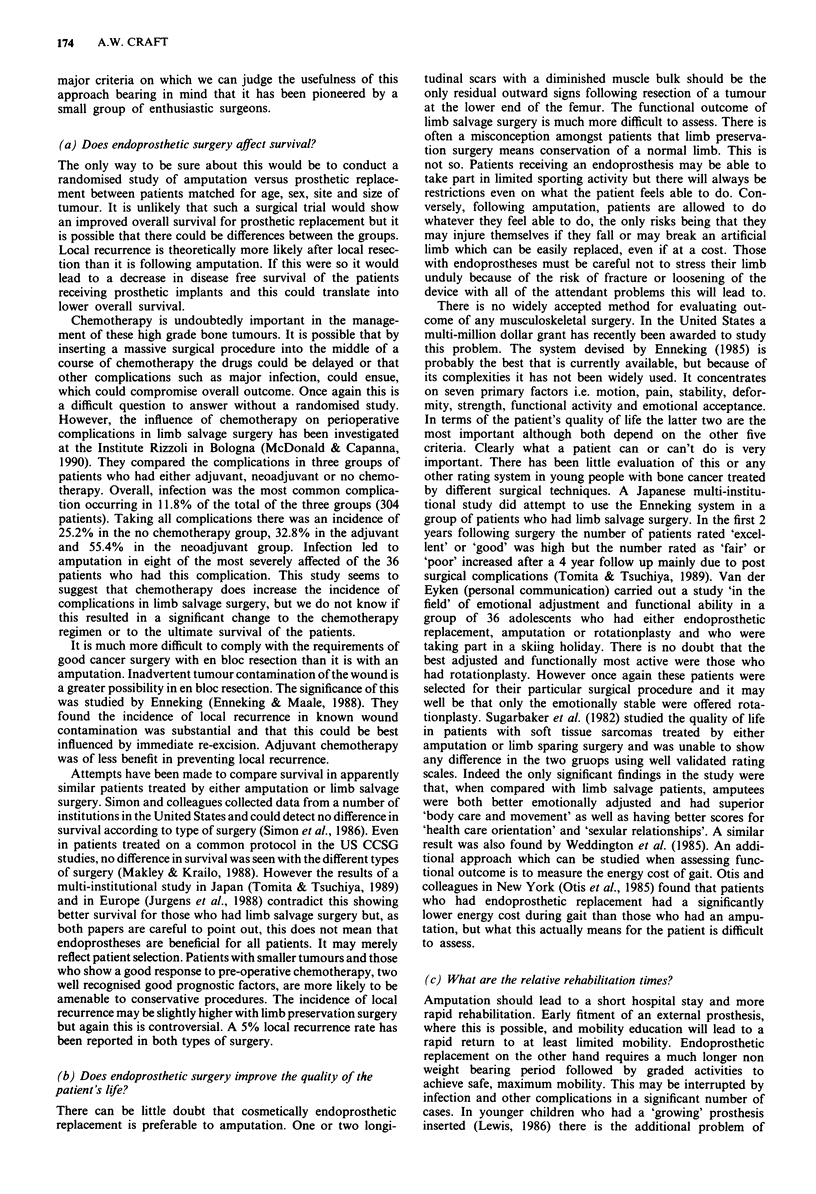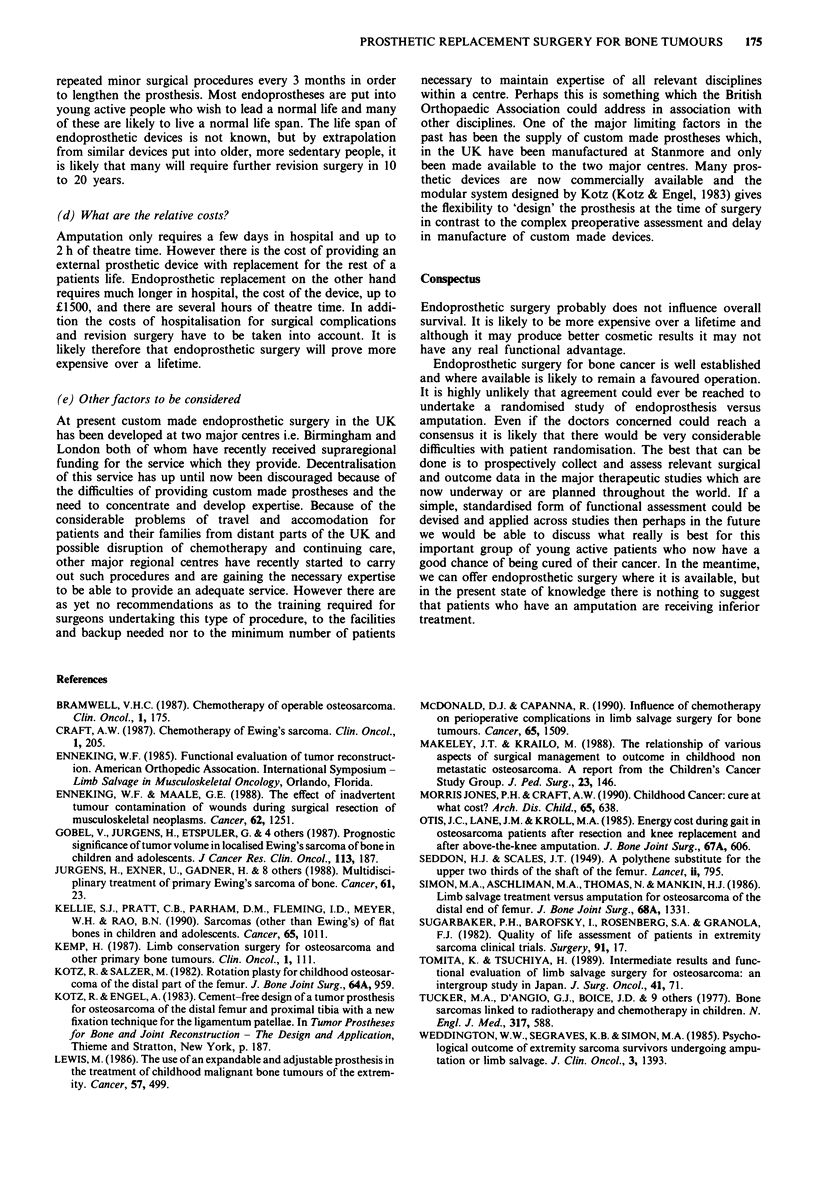# Prosthetic replacement surgery for bone tumours--cure at less cost?

**DOI:** 10.1038/bjc.1991.42

**Published:** 1991-02

**Authors:** A. W. Craft


Br. J. Cancer (1991), 63, 173–175 © Macmillan Press Ltd., 1991

GUEST EDITORIAL

### Prosthetic replacement surgery for bone tumours – cure at less cost?

A.W. Craft

*Department of Child Health, The Medical School, Framlington Place, Newcastle-upon-Tyne NE2 4HH, UK.*

The dramatic improvements which have occurred over the past 20 years in the management of children and young people with cancer, along with the recognition of significant late effects of treatment, has led to a reappraisal of the overall philosophy of management. At a time when most children died of their disease a policy of cure 'at any cost' was justified. Now we have moved into an era of cure 'at least cost' (Morris Jones & Craft, 1990) and this is well exemplified by the management of bone tumours in young people. Indeed the 'Cade' approach to the management of osteosarcoma, namely radiotherapy to the primary tumour followed by delayed amputation if the patient had not developed metastases, could be said to be a forerunner of this philosophy in that its intention was to 'manage' at least cost.

Osteosarcoma and Ewing's tumour are the two common malignant primary bone tumours which occur in children and young adults with a peak incidence for both in the teenage years. It is at this time of life that amputation can be so devastating to a growing child with an everburgeoning perception of their own body image. Amputation in this age group is perhaps one of the most distressing forms of treatment which doctors and nurses have to inflict on patients and it is not surprising that alternative methods of therapy have been sought. Limb salvage surgery therefore has developed over the last 40 years in an attempt to mitigate the effects of this mutilating surgery. In addition to en bloc resection and replacement with a metal prosthesis other limb salvage methods have also been developed. Amongst these is rotation plasty (Kotz & Salzer, 1982), widely used for lower femoral osteosarcoma in the remainder of Europe, but less so in the UK, which consists of resection of the tumour bone and its surrounding structure followed by rotation of the tibia through 180° and its reimplantation on the remains of the femur. The ankle then becomes the knee and the artificial limb is attached to the foot. Critics of this form of surgery suggest severe psychological distrubance may ensue but this does not seem to be the case. Many consider it a modified amputation.

The primary aim of treatment for any cancer must be to cure the patient if at all possible. In the pre-chemotherapy era, only 20% of patients with osteosarcoma were cured by surgery alone and without chemotherapy or radiotherapy virtually all patients with Ewing's sarcoma died. Death was usually due to the development of distant metastases. The advent of chemotherapy has resulted in a dramatic improvement in survival for osteosarcoma patients to between 50 and 60% in most multi-institutional studies (Bramwell, 1987). Chemotherapy and radiotherapy have led to a reported 50% survival rate for Ewing's sarcoma (Craft, 1987). The treatment for both tumour types is in two phases, one being the definitive treatment of the primary tumour by surgery, radiotherapy or both and the other is the treatment of covert micrometastatic disease by chemotherapy. The latter usually also has some effect on the primary tumour. The definitive treatment of the primary tumour in osteogenic sarcoma must be surgery if complete ablation is to succeed, whereas this is often possible with a combination of chemotherapy and radiotherapy in Ewing's tumour. It is unlikely that, with the present chemotherapeutic agents, osteosarcoma will ever be cured without surgery. A recent report of 'flat bone' primary osteosarcoma showed that only where complete surgical excision was possible could cure be obtained (Kellie *et al.*, 1990). Although Ewing's tumour can be locally controlled by radiotherapy this modality does have significant late effects including radiation necrosis of bone with the risk of fracture, contraction of soft tissue with tendon shortening and the possibility of second malignancy (Tucker *et al.*, 1977). Because of these factors surgery has been employed more often and recently been shown to be a safe, and perhaps even a prognosticially beneficial option in the management of Ewing's tumour (Gobel *et al.*, 1987). Surgery should be considered mandatory in treating osteosarcoma and, although optional, may have some significant benefits in managing Ewing's tumour.

If surgery is contemplated is limb salvage with prosthetic replacement the preferred option? It is now more than 40 years since the first prosthetic replacement for a bone tumour was carried out in the UK (Seddon & Scales, 1949) using a hand carved 'polythene' prosthesis. Since then prostheses production and their surgical implantation has grown rapidly. Much of the knowledge of the prosthetic devices has been a consequence of the experience in arthroplasty performed for arthritic joints but the surgery itself has had to be developed specifically with cancer in mind. That the surgery can be successful is without doubt. Between 1949 and 1986 680 custom made prostheses were manufactured and inserted at the two major national bone tumour centres in the UK in London and Birmingham. Most of these were for cancer and many patients have been long term survivors with a functioning prosthesis (Kemp, 1987).

In Europe the major group carrying out co-operative studies in Ewing's tumour is the German CESS (Cooperative Ewing's Sarcoma Study) group. In CESS 81 surgery for local control was carried out in 60 (65%) of 93 patients, 14 (15%) of these being extracompartmental resections rather than amputation (Jurgens *et al.*, 1988). In CESS 86 74% of patients had primary surgery, 67% of these having resection rather than amputation (H. Jurgens, personal communication). In the UK the MRC/UKCCSG have been involved in the European Osteosarcoma Intergroup (EOI) studies for osteosarcoma where there has again been an increasing use of conservative surgery from 62% in the 80831 study to 70% in 80861 (MRC unpublished data). Endoprosthetic replacement surgery therefore has gained an increasing place in the management of bone tumours in young people.

Clinicians working in this field can confidently expect some patients to walk into the room following a prosthetic replacement without any significant disability and to be leading a virtually normal life. However they also see some patients in whom there have been significant complications which may even result in eventual amputation. The media are also constantly showing us the incredible achievements of some of those who have had an amputation, because often these patients achieve more on one leg than most people achieve with two legs. Has prosthetic implant therefore been worthwhile and is it better than the alternative of amputation or for, Ewing's tumour, irradiation? It is worth assessing the

Received 3 August 1990; and in revised form 18 September 1990.

major criteria on which we can judge the usefulness of this approach bearing in mind that it has been pioneered by a small group of enthusiastic surgeons.

*(a) Does endoprosthetic surgery affect survival?*

The only way to be sure about this would be to conduct a randomised study of amputation versus prosthetic replacement between patients matched for age, sex, site and size of tumour. It is unlikely that such a surgical trial would show an improved overall survival for prosthetic replacement but it is possible that there could be differences between the groups. Local recurrence is theoretically more likely after local resection than it is following amputation. If this were so it would lead to a decrease in disease free survival of the patients receiving prosthetic implants and this could translate into lower overall survival.

Chemotherapy is undoubtedly important in the management of these high grade bone tumours. It is possible that by inserting a massive surgical procedure into the middle of a course of chemotherapy the drugs could be delayed or that other complications such as major infection, could ensue, which could compromise overall outcome. Once again this is a difficult question to answer without a randomised study. However, the influence of chemotherapy on perioperative complications in limb salvage surgery has been investigated at the Institute Rizzoli in Bologna (McDonald & Capanna, 1990). They compared the complications in three groups of patients who had either adjuvant, neoadjuvant or no chemotherapy. Overall, infection was the most common complication occurring in 11.8% of the total of the three groups (304 patients). Taking all complications there was an incidence of 25.2% in the no chemotherapy group, 32.8% in the adjuvant and 55.4% in the neoadjuvant group. Infection led to amputation in eight of the most severely affected of the 36 patients who had this complication. This study seems to suggest that chemotherapy does increase the incidence of complications in limb salvage surgery, but we do not know if this resulted in a significant change to the chemotherapy regimen or to the ultimate survival of the patients.

It is much more difficult to comply with the requirements of good cancer surgery with en bloc resection than it is with an amputation. Inadvertent tumour contamination of the wound is a greater possibility in en bloc resection. The significance of this was studied by Enneking (Enneking & Maale, 1988). They found the incidence of local recurrence in known wound contamination was substantial and that this could be best influenced by immediate re-excision. Adjuvant chemotherapy was of less benefit in preventing local recurrence.

Attempts have been made to compare survival in apparently similar patients treated by either amputation or limb salvage surgery. Simon and colleagues collected data from a number of institutions in the United States and could detect no difference in survival according to type of surgery (Simon *et al.*, 1986). Even in patients treated on a common protocol in the US CCSG studies, no difference in survival was seen with the different types of surgery (Makley & Krailo, 1988). However the results of a multi-institutional study in Japan (Tomita & Tsuchiya, 1989) and in Europe (Jurgens *et al.*, 1988) contradict this showing better survival for those who had limb salvage surgery but, as both papers are careful to point out, this does not mean that endoprostheses are beneficial for all patients. It may merely reflect patient selection. Patients with smaller tumours and those who show a good response to pre-operative chemotherapy, two well recognised good prognostic factors, are more likely to be amenable to conservative procedures. The incidence of local recurrence may be slightly higher with limb preservation surgery but again this is controversial. A 5% local recurrence rate has been reported in both types of surgery.

*(b) Does endoprosthetic surgery improve the quality of the patient's life?*

There can be little doubt that cosmetically endoprosthetic replacement is preferable to amputation. One or two longitudinal scars with a diminished muscle bulk should be the only residual outward signs following resection of a tumour at the lower end of the femur. The functional outcome of limb salvage surgery is much more difficult to assess. There is often a misconception amongst patients that limb preservation surgery means conservation of a normal limb. This is not so. Patients receiving an endoprosthesis may be able to take part in limited sporting activity but there will always be restrictions even on what the patient feels able to do. Conversely, following amputation, patients are allowed to do whatever they feel able to do, the only risks being that they may injure themselves if they fall or may break an artificial limb which can be easily replaced, even if at a cost. Those with endoprostheses must be careful not to stress their limb unduly because of the risk of fracture or loosening of the device with all of the attendant problems this will lead to.

There is no widely accepted method for evaluating outcome of any musculoskeletal surgery. In the United States a multi-million dollar grant has recently been awarded to study this problem. The system devised by Enneking (1985) is probably the best that is currently available, but because of its complexities it has not been widely used. It concentrates on seven primary factors i.e. motion, pain, stability, deformity, strength, functional activity and emotional acceptance. In terms of the patient's quality of life the latter two are the most important although both depend on the other five criteria. Clearly what a patient can or can't do is very important. There has been little evaluation of this or any other rating system in young people with bone cancer treated by different surgical techniques. A Japanese multi-institutional study did attempt to use the Enneking system in a group of patients who had limb salvage surgery. In the first 2 years following surgery the number of patients rated 'excellent' or 'good' was high but the number rated as 'fair' or 'poor' increased after a 4 year follow up mainly due to post surgical complications (Tomita & Tsuchiya, 1989). Van der Eyken (personal communication) carried out a study 'in the field' of emotional adjustment and functional ability in a group of 36 adolescents who had either endoprosthetic replacement, amputation or rotationplasty and who were taking part in a skiing holiday. There is no doubt that the best adjusted and functionally most active were those who had rotationplasty. However once again these patients were selected for their particular surgical procedure and it may well be that only the emotionally stable were offered rotationplasty. Sugarbaker *et al.* (1982) studied the quality of life in patients with soft tissue sarcomas treated by either amputation or limb sparing surgery and was unable to show any difference in the two gruops using well validated rating scales. Indeed the only significant findings in the study were that, when compared with limb salvage patients, amputees were both better emotionally adjusted and had superior 'body care and movement' as well as having better scores for 'health care orientation' and 'sexular relationships'. A similar result was also found by Weddington *et al.* (1985). An additional approach which can be studied when assessing functional outcome is to measure the energy cost of gait. Otis and colleagues in New York (Otis *et al.*, 1985) found that patients who had endoprosthetic replacement had a significantly lower energy cost during gait than those who had an amputation, but what this actually means for the patient is difficult to assess.

*(c) What are the relative rehabilitation times?*

Amputation should lead to a short hospital stay and more rapid rehabilitation. Early fitment of an external prosthesis, where this is possible, and mobility education will lead to a rapid return to at least limited mobility. Endoprosthetic replacement on the other hand requires a much longer non weight bearing period followed by graded activities to achieve safe, maximum mobility. This may be interrupted by infection and other complications in a significant number of cases. In younger children who had a 'growing' prosthesis inserted (Lewis, 1986) there is the additional problem of repeated minor surgical procedures every 3 months in order to lengthen the prosthesis. Most endoprostheses are put into young active people who wish to lead a normal life and many of these are likely to live a normal life span. The life span of endoprosthetic devices is not known, but by extrapolation from similar devices put into older, more sedentary people, it is likely that many will require further revision surgery in 10 to 20 years.

*(d) What are the relative costs?*

Amputation only requires a few days in hospital and up to 2 h of theatre time. However there is the cost of providing an external prosthetic device with replacement for the rest of a patients life. Endoprosthetic replacement on the other hand requires much longer in hospital, the cost of the device, up to £1500, and there are several hours of theatre time. In addition the costs of hospitalisation for surgical complications and revision surgery have to be taken into account. It is likely therefore that endoprosthetic surgery will prove more expensive over a lifetime.

*(e) Other factors to be considered*

At present custom made endoprosthetic surgery in the UK has been developed at two major centres i.e. Birmingham and London both of whom have recently received supraregional funding for the service which they provide. Decentralisation of this service has up until now been discouraged because of the difficulties of providing custom made prostheses and the need to concentrate and develop expertise. Because of the considerable problems of travel and accomodation for patients and their families from distant parts of the UK and possible disruption of chemotherapy and continuing care, other major regional centres have recently started to carry out such procedures and are gaining the necessary expertise to be able to provide an adequate service. However there are as yet no recommendations as to the training required for surgeons undertaking this type of procedure, to the facilities and backup needed nor to the minimum number of patients necessary to maintain expertise of all relevant disciplines within a centre. Perhaps this is something which the British Orthopaedic Association could address in association with other disciplines. One of the major limiting factors in the past has been the supply of custom made prostheses which, in the UK have been manufactured at Stanmore and only been made available to the two major centres. Many prosthetic devices are now commercially available and the modular system designed by Kotz (Kotz & Engel, 1983) gives the flexibility to 'design' the prosthesis at the time of surgery in contrast to the complex preoperative assessment and delay in manufacture of custom made devices.

### Conspectus

Endoprosthetic surgery probably does not influence overall survival. It is likely to be more expensive over a lifetime and although it may produce better cosmetic results it may not have any real functional advantage.

Endoprosthetic surgery for bone cancer is well established and where available is likely to remain a favoured operation. It is highly unlikely that agreement could ever be reached to undertake a randomised study of endoprosthesis versus amputation. Even if the doctors concerned could reach a consensus it is likely that there would be very considerable difficulties with patient randomisation. The best that can be done is to prospectively collect and assess relevant surgical and outcome data in the major therapeutic studies which are now underway or are planned throughout the world. If a simple, standardised form of functional assessment could be devised and applied across studies then perhaps in the future we would be able to discuss what really is best for this important group of young active patients who now have a good chance of being cured of their cancer. In the meantime, we can offer endoprosthetic surgery where it is available, but in the present state of knowledge there is nothing to suggest that patients who have an amputation are receiving inferior treatment.

### References

BRAMWELL, V.H.C. (1987). Chemotherapy of operable osteosarcoma. *Clin. Oncol.*, **1,** 175.

CRAFT, A.W. (1987). Chemotherapy of Ewing's sarcoma. *Clin. Oncol.*, **1,** 205.

ENNEKING, W.F. (1985). Functional evaluation of tumor reconstruction. American Orthopedic Assocation. International Symposium – *Limb Salvage in Musculoskeletal Oncology*, Orlando, Florida.

ENNEKING, W.F. & MAALE, G.E. (1988). The effect of inadvertent tumour contamination of wounds during surgical resection of musculoskeletal neoplasms. *Cancer*, **62,** 1251.

GOBEL, V., JURGENS, H., ETSPULER, G. & 4 others (1987). Prognostic significance of tumor volume in localised Ewing's sarcoma of bone in children and adolescents. *J Cancer Res. Clin. Oncol.*, **113,** 187.

JURGENS, H., EXNER, U., GADNER, H. & 8 others (1988). Multidisciplinary treatment of primary Ewing's sarcoma of bone. *Cancer*, **61,** 23.

KELLIE, S.J., PRATT, C.B., PARHAM, D.M., FLEMING, I.D., MEYER, W.H. & RAO, B.N. (1990). Sarcomas (other than Ewing's) of flat bones in children and adolescents. *Cancer*, **65,** 1011.

KEMP, H. (1987). Limb conservation surgery for osteosarcoma and other primary bone tumours. *Clin. Oncol.*, **1,** 111.

KOTZ, R. & SALZER, M. (1982). Rotation plasty for childhood osteosarcoma of the distal part of the femur. *J. Bone Joint Surg.*, **64A,** 959.

KOTZ, R. & ENGEL, A. (1983). Cement-free design of a tumor prosthesis for osteosarcoma of the distal femur and proximal tibia with a new fixation technique for the ligamentum patellae. In *Tumor Prostheses for Bone and Joint Reconstruction – The Design and Application*, Thieme and Stratton, New York, p. 187.

LEWIS, M. (1986). The use of an expandable and adjustable prosthesis in the treatment of childhood malignant bone tumours of the extremity. *Cancer*, **57,** 499.

MCDONALD, D.J. & CAPANNA, R. (1990). Influence of chemotherapy on perioperative complications in limb salvage surgery for bone tumours. *Cancer*, **65,** 1509.

MAKELEY, J.T. & KRAILO, M. (1988). The relationship of various aspects of surgical management to outcome in childhood non metastatic osteosarcoma. A report from the Children's Cancer Study Group. *J. Ped. Surg.*, **23,** 146.

MORRIS JONES, P.H. & CRAFT, A.W. (1990). Childhood Cancer: cure at what cost? *Arch. Dis. Child.*, **65,** 638.

OTIS, J.C., LANE, J.M. & KROLL, M.A. (1985). Energy cost during gait in osteosarcoma patients after resection and knee replacement and after above-the-knee amputation. *J. Bone Joint Surg.*, **67A,** 606.

SEDDON, H.J. & SCALES, J.T. (1949). A polythene substitute for the upper two thirds of the shaft of the femur. *Lancet*, **ii,** 795.

SIMON, M.A., ASCHLIMAN, M.A., THOMAS, N. & MANKIN, H.J. (1986). Limb salvage treatment versus amputation for osteosarcoma of the distal end of femur. *J. Bone Joint Surg.*, **68A,** 1331.

SUGARBAKER, P.H., BAROFSKY, I., ROSENBERG, S.A. & GRANOLA, F.J. (1982). Quality of life assessment of patients in extremity sarcoma clinical trials. *Surgery*, **91,** 17.

TOMITA, K. & TSUCHIYA, H. (1989). Intermediate results and functional evaluation of limb salvage surgery for osteosarcoma: an intergroup study in Japan. *J. Surg. Oncol.*, **41,** 71.

TUCKER, M.A., D'ANGIO, G.J., BOICE, J.D. & 9 others (1977). Bone sarcomas linked to radiotherapy and chemotherapy in children. *N. Engl. J. Med.*, **317,** 588.

WEDDINGTON, W.W., SEGRAVES, K.B. & SIMON, M.A. (1985). Psychological outcome of extremity sarcoma survivors undergoing amputation or limb salvage. *J. Clin. Oncol.*, **3,** 1393.